# Supplementary material for: Bioactive Constituents from the Roots of Eurycoma longifolia
Source: Molecules. 2019 Aug 30;24(17):3157. doi: 10.3390/molecules24173157 (PMC6749187; doi:10.3390/molecules24173157)
Supplement: Supplementary file 1 [file molecules-24-03157-s001.pdf]

Supporting Information

Bioactive Constituents from the Roots of *Eurycoma longifolia*

JingYa Ruan [1,¶], Zheng Li [1,¶], Ying Zhang [2], Yue Chen [2], Mengyang Liu [2], Lifeng Han [1], Yi Zhang [1,2,*] and Tao Wang [1,2,*]

[1] Tianjin State Key Laboratory of Modern Chinese Medicine, 312 Anshanxi Road, Nankai District, Tianjin 300193, China; Ruanjy19930919@163.com (J.R.); wo15510977612@163.com (Z.L.); hanlifeng_1@sohu.com (L.H.)

[2] Tianjin Key Laboratory of TCM Chemistry and Analysis, Institute of Traditional Chinese Medicine, Tianjin University of Traditional Chinese Medicine, 312 Anshanxi Road, Nankai District, Tianjin 300193, China; zyingtzy@163.com (Y.Z.); YueChen17208@gmail.com (Y.C.); liumengyang0212@tjutcm.edu.cn (M.L.)

¶ J. Ruan and Z. Li contributed equally to this work.

* Correspondence: zhwwxzh@tjutcm.edu.cn (Y.Z.); wangtao@tjutcm.edu.cn (T.W.); Tel./Fax: +86-22-5959-6168 (T.W.)

Figure S1 ^{1}H NMR (600 MHz, C_5D_5N) spectrum of compound **1**. 3
Figure S2 ^{13}C NMR (150 MHz, C_5D_5N) spectrum of compound **1**. 3
Figure S3 ^{1}H ^{1}H COSY (C_5D_5N) spectrum of compound **1**. 4
Figure S4 HSQC (C_5D_5N) spectrum of compound **1**. 4
Figure S5 HMBC (C_5D_5N) spectrum of compound **1**. 5
Figure S6 NOESY (C_5D_5N) spectrum of compound **1**. 5
Figure S7 HRESI-TOF-MS spectrum of compound **1**. 6
Figure S8 ^{1}H NMR (500 MHz, C_5D_5N) spectrum of compound **2**. 7
Figure S9 ^{13}C NMR (125 MHz, C_5D_5N) spectrum of compound **2**. 7
Figure S10 DEPT 135 (C_5D_5N) spectrum of compound **2**. 8
Figure S11 ^{1}H ^{1}H COSY (C_5D_5N) spectrum of compound **2**. 8
Figure S12 HSQC (C_5D_5N) spectrum of compound **2**. 9
Figure S13 HMBC (C_5D_5N) spectrum of compound **2**. 9
Figure S14 HRESI-TOF-MS spectrum of compound **2**. 10
Figure S15 ^{1}H NMR (500 MHz, CD_3OD) spectrum of compound **3**. 11
Figure S16 ^{13}C NMR (125 MHz, CD_3OD) spectrum of compound **3**. 11
Figure S17 DEPT 135 (CD_3OD) spectrum of compound **3**. 12
Figure S18 ^{1}H ^{1}H COSY (C_5D_5N) spectrum of compound **3**. 12
Figure S19 HSQC (C_5D_5N) spectrum of compound **3**. 13
Figure S20 HMBC (C_5D_5N) spectrum of compound **3**. 13
Figure S21 HRESI-TOF-MS spectrum of compound **3**. 14
Figure S22 ^{1}H NMR (500 MHz, CD_3OD) spectrum of compound **4**. 15
Figure S23 ^{13}C NMR (125 MHz, CD_3OD) spectrum of compound **4**. 15
Figure S24 DEPT 135 (CD_3OD) spectrum of compound **4**. 16
Figure S25 ^{1}H ^{1}H COSY (CD_3OD) spectrum of compound **4**. 16
Figure S26 HSQC (CD_3OD) spectrum of compound **4**. 17
Figure S27 HMBC (CD_3OD) spectrum of compound **4**. 17
Figure S28 HRESI-TOF-MS spectrum of compound **4**. 18
Figure S29 MTT analysis of **1–16** obtained from *Cortex Dictamni* on RAW264.7 cells. 19
Figure S30 Raw quantification data for figure 6 20
Figure S31 Raw quantification data for figure 7 20
Figure S32 Raw quantification data for figure 8 21
Figure S33 Raw quantification data for figure 9 21

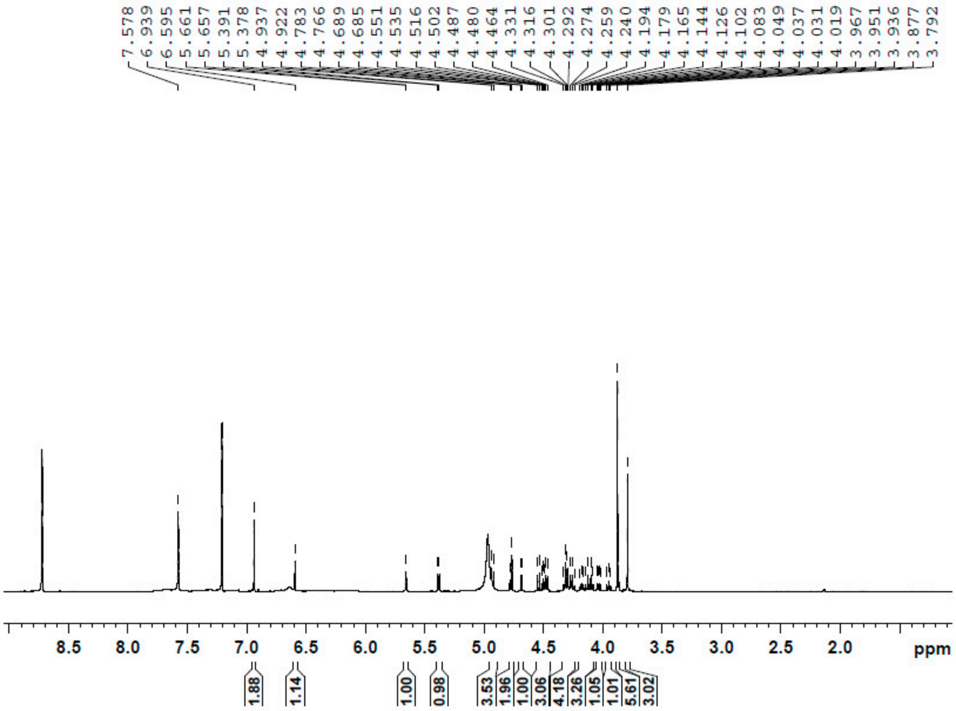

Figure S1 ^{1}H NMR (600 MHz, C_5D_5N) spectrum of compound **1**

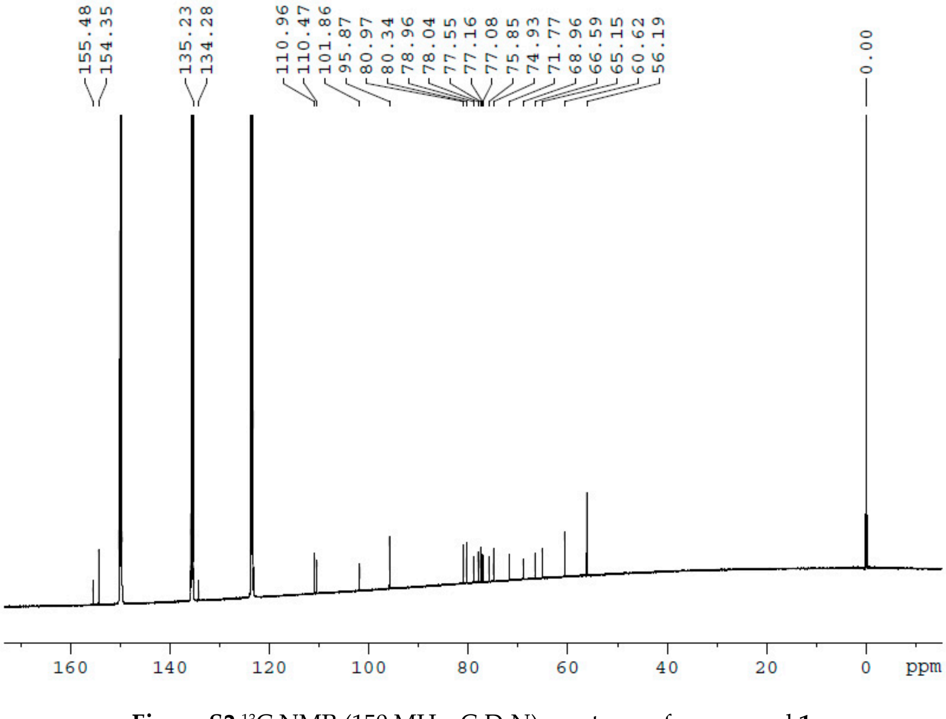

Figure S2 ^{13}C NMR (150 MHz, C_5D_5N) spectrum of compound **1**

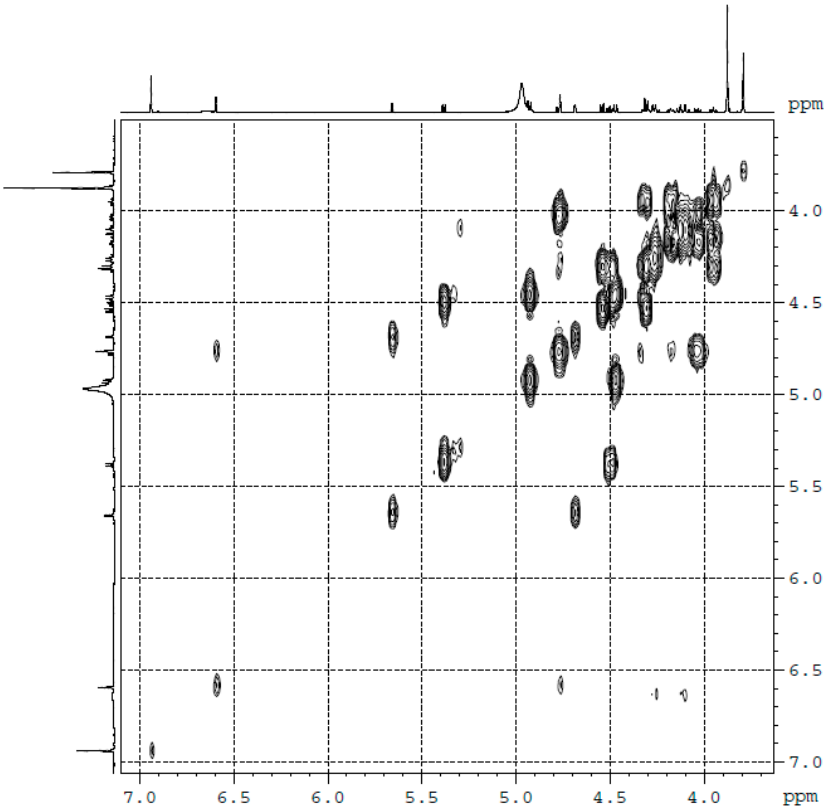

Figure S3 ¹H ¹H COSY (C_5D_5N) spectrum of compound **1**

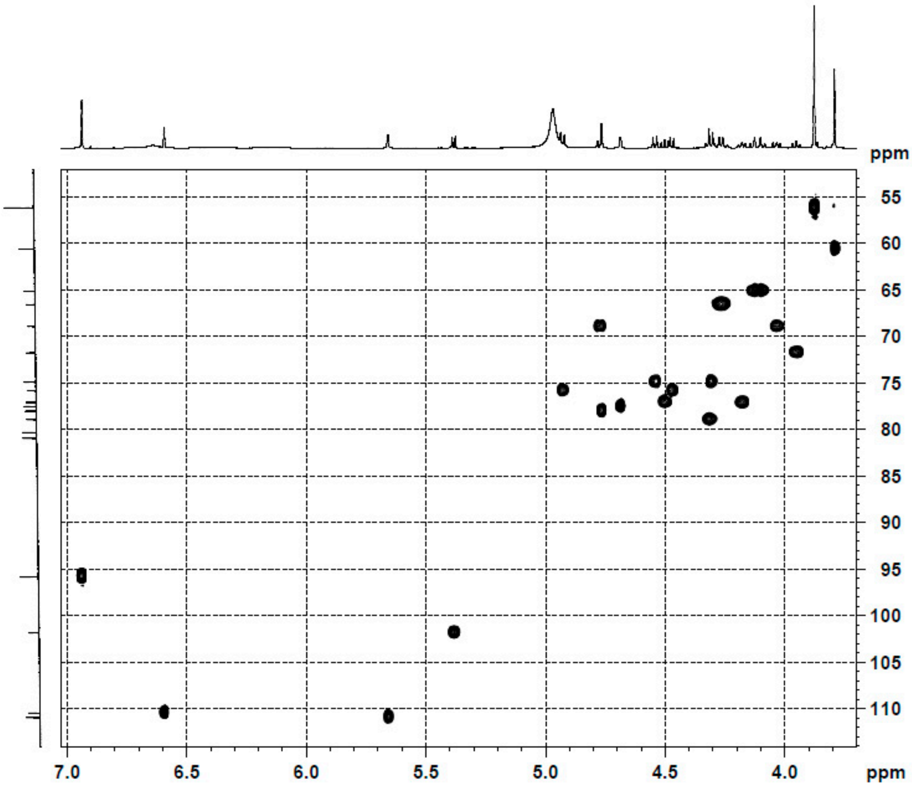

Figure S4 HSQC (C_5D_5N) spectrum of compound **1**

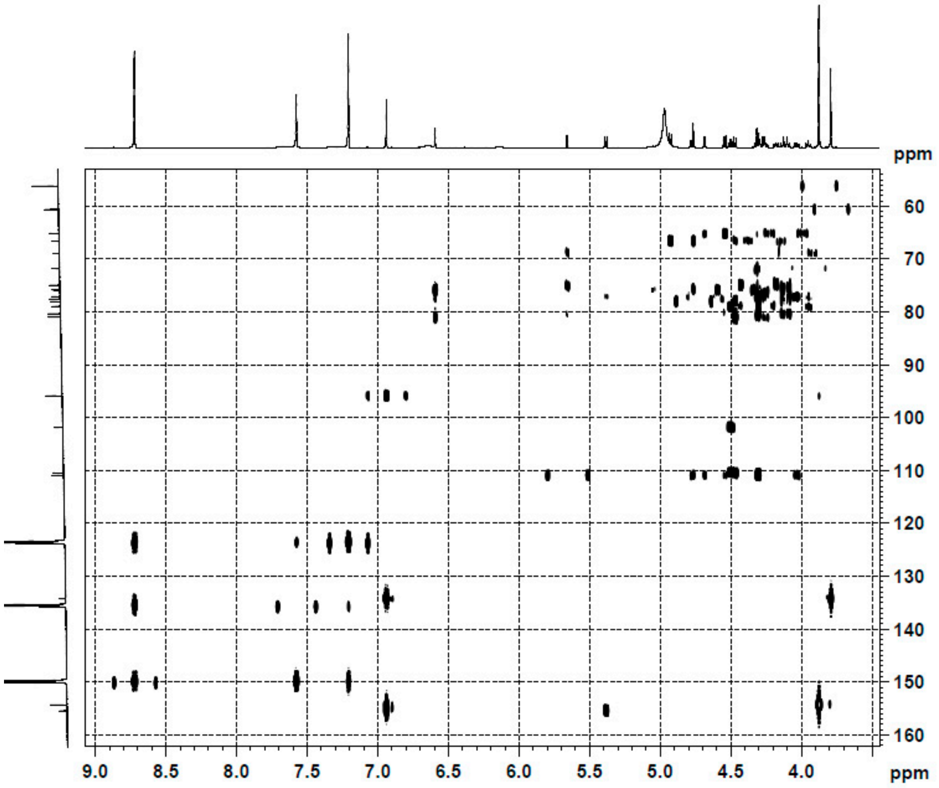

Figure S5 HMBC (C_5D_5N) spectrum of compound **1**

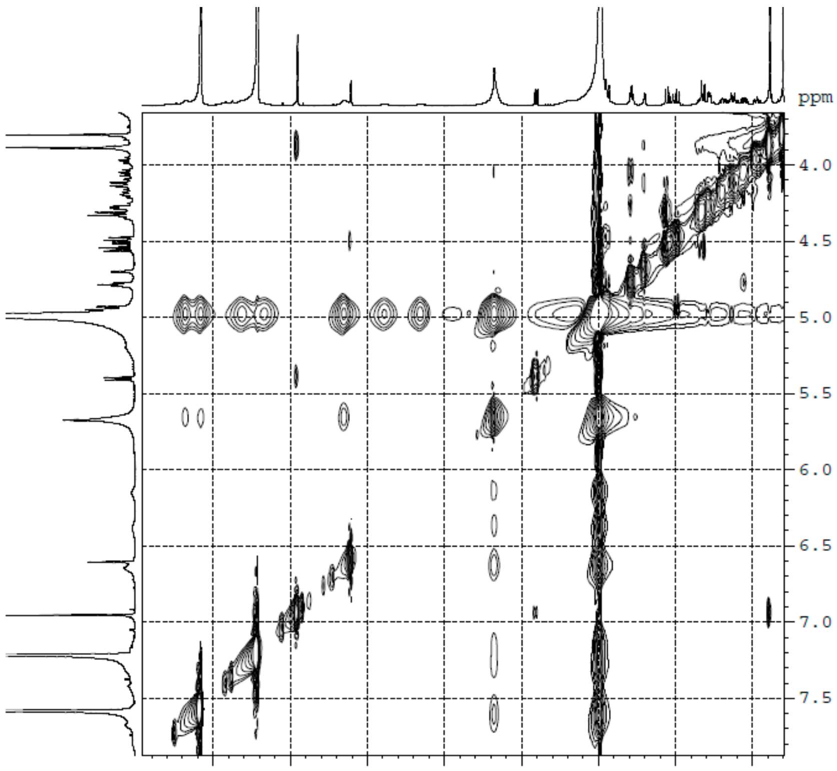

Figure S6 NOESY (C_5D_5N) spectrum of compound **1**

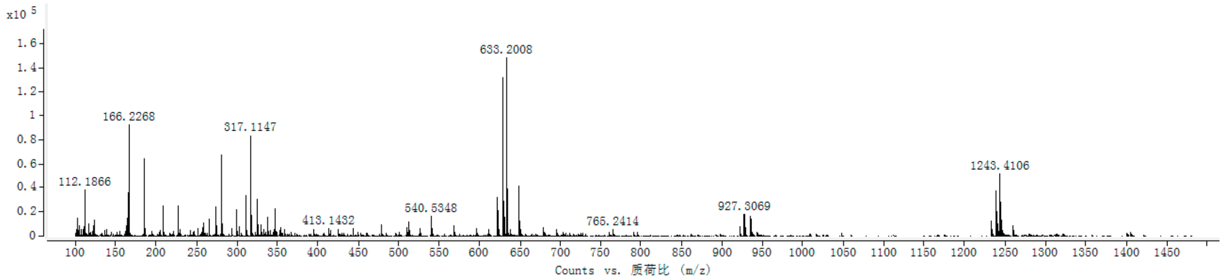

Figure S7 HRESI-TOF-MS spectrum of compound **1**

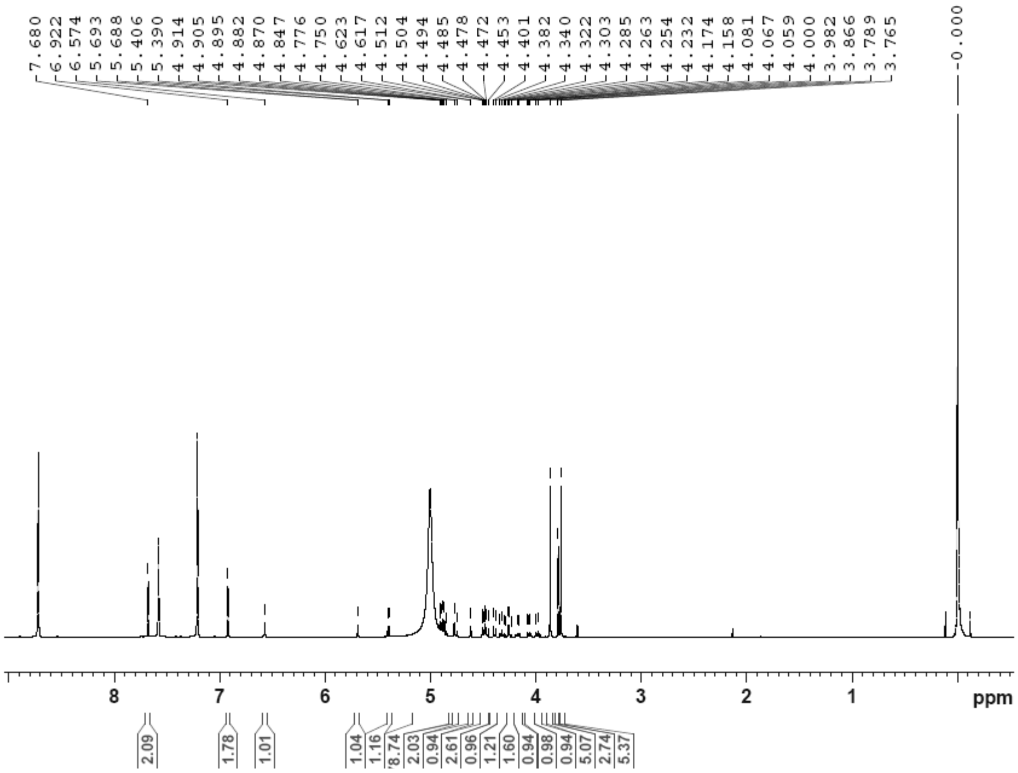

Figure S8 ^{1}H NMR (500 MHz, C_5D_5N) spectrum of compound **2**

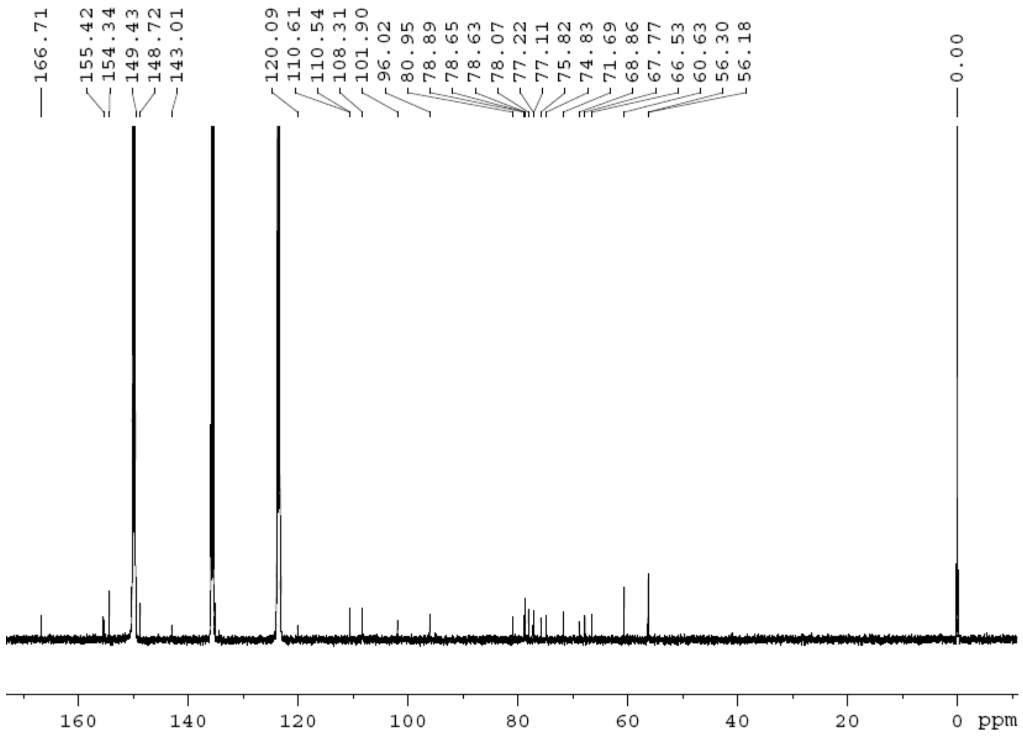

Figure S9 ^{13}C NMR (125 MHz, C_5D_5N) spectrum of compound **2**

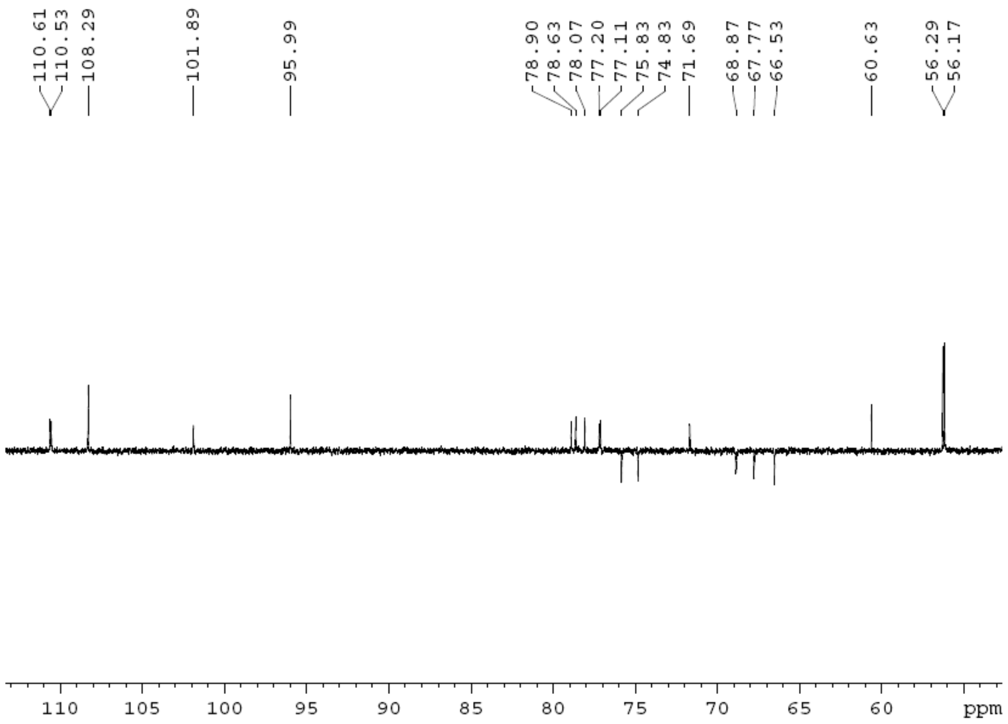

Figure S10 DEPT 135 (C_5D_5N) spectrum of compound **2**

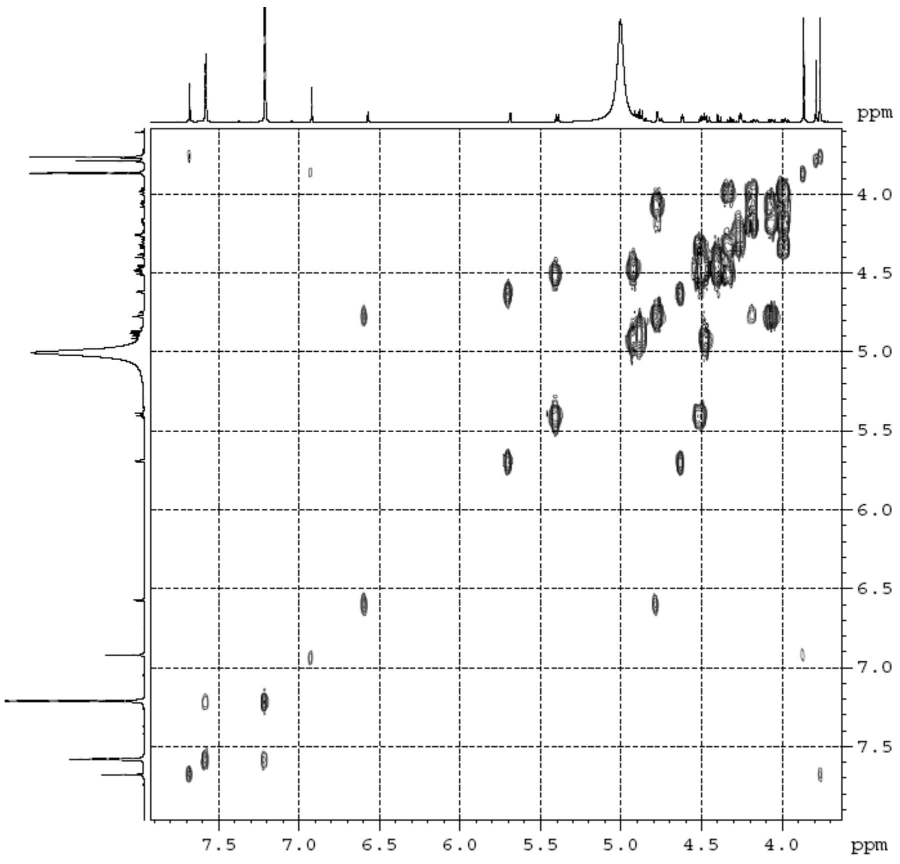

Figure S11 ^{1}H ^{1}H COSY (C_5D_5N) spectrum of compound **2**

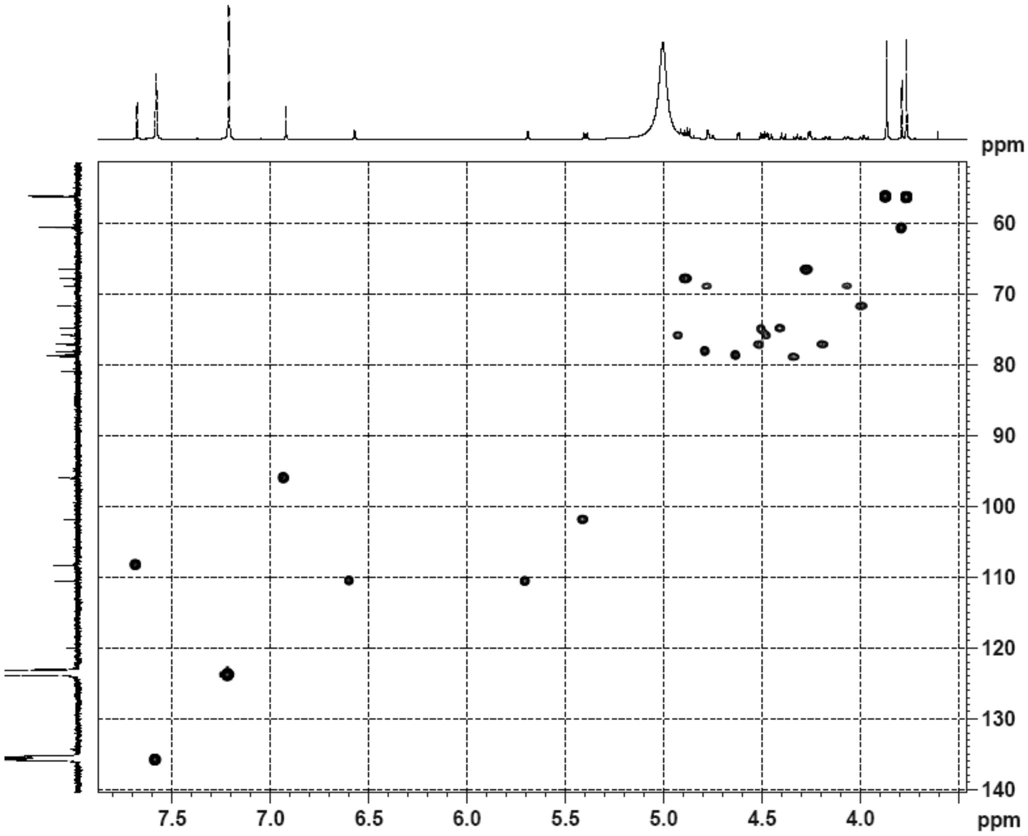

Figure S12 HSQC (C_5D_5N) spectrum of compound **2**

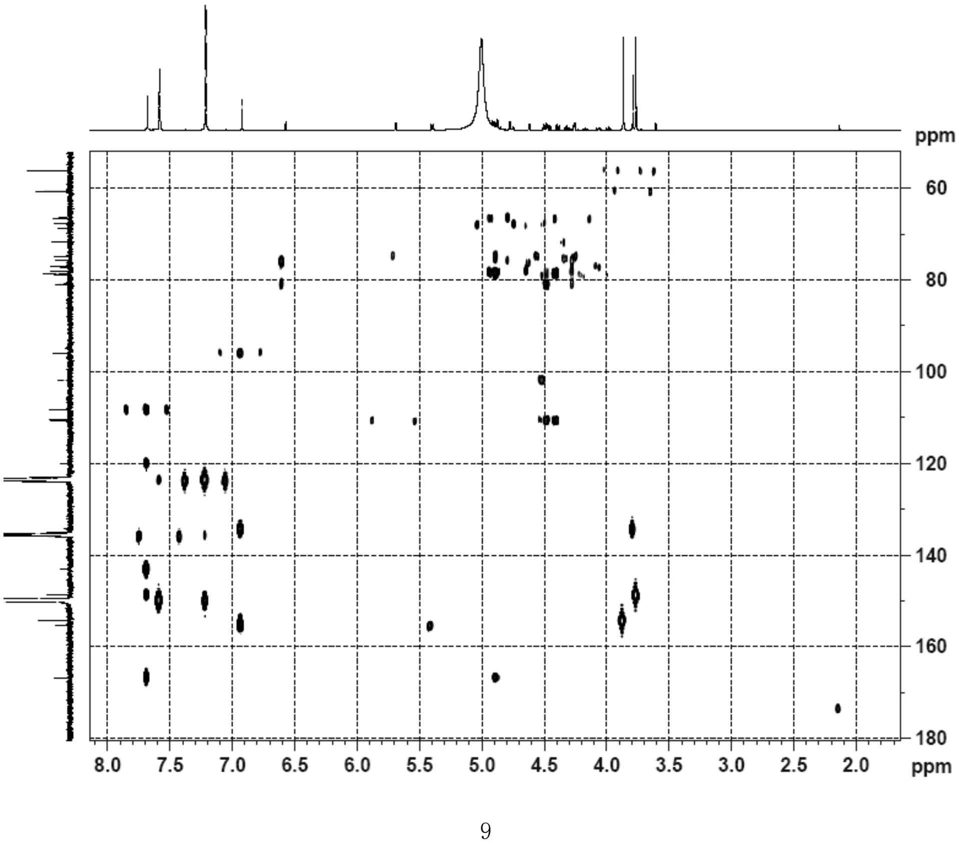

Figure S13 HMBC (C_5D_5N) spectrum of compound **2**

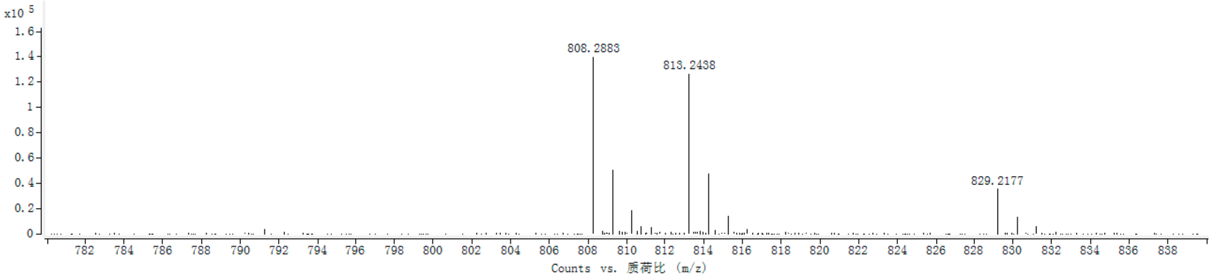

Figure S14 HRESI-TOF-MS spectrum of compound **2**

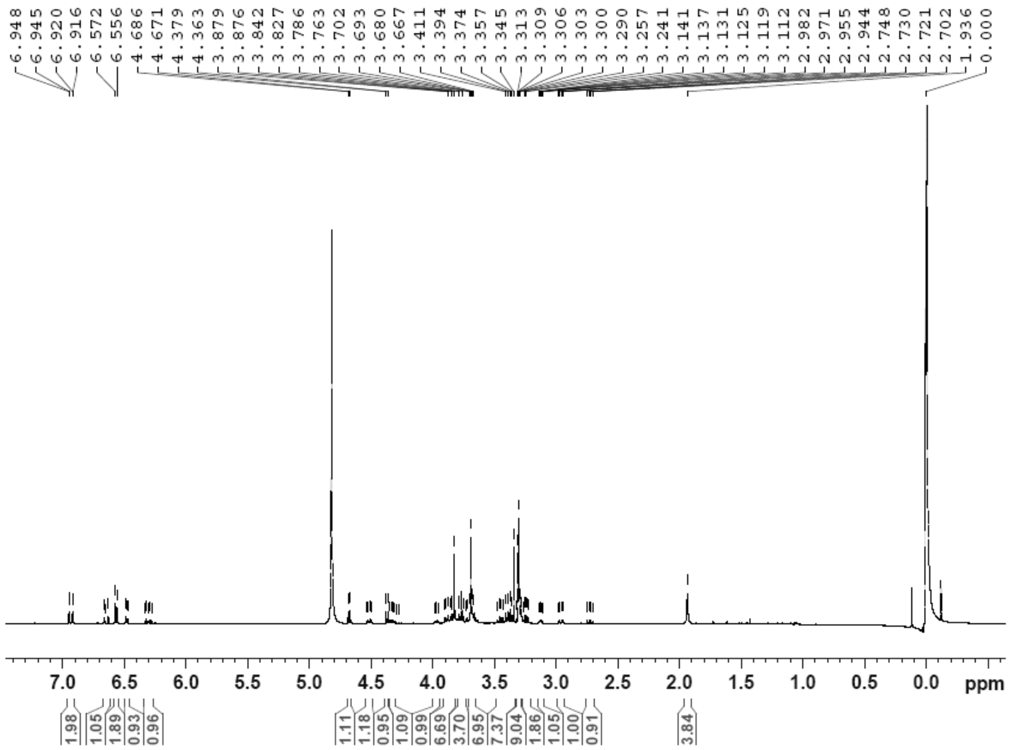

Figure S15 ^{1}H NMR (500 MHz, CD_3OD) spectrum of compound **3**

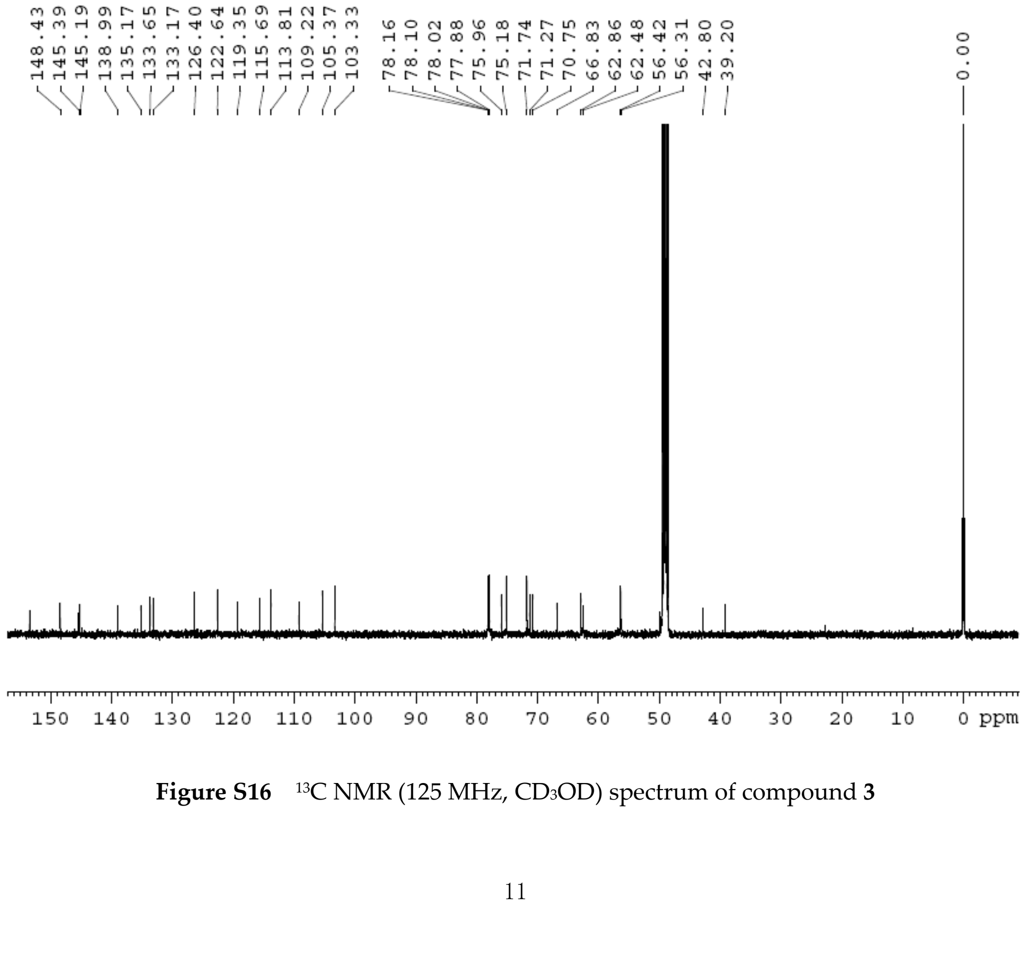

Figure S16 ^{13}C NMR (125 MHz, CD_3OD) spectrum of compound **3**

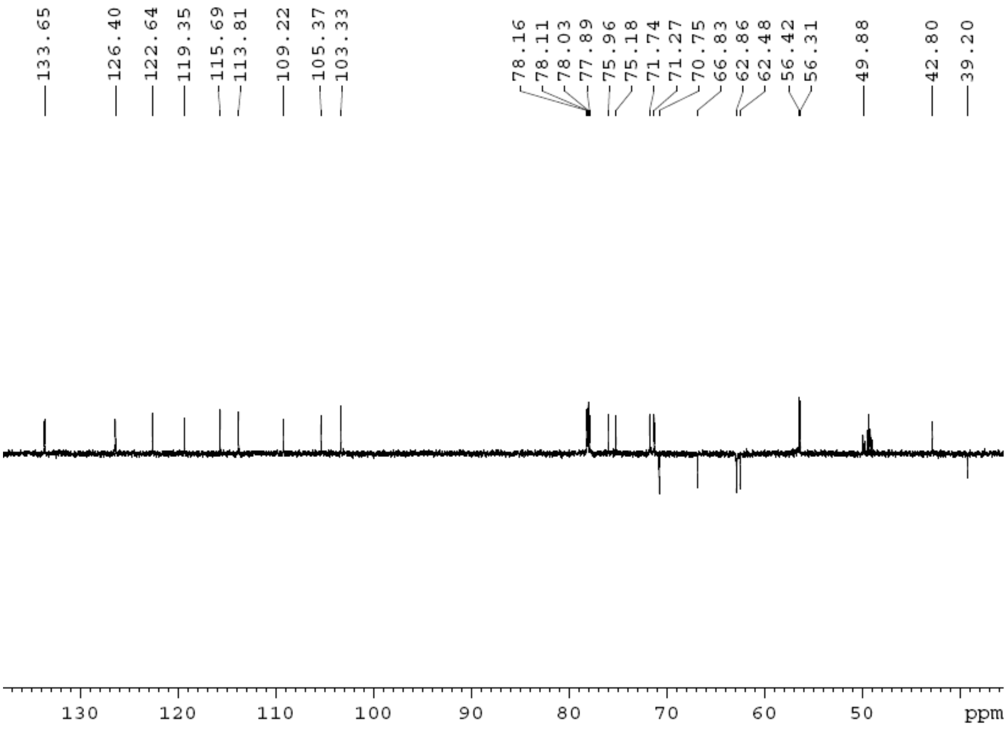

Figure S17 DEPT 135 (CD_3OD) spectrum of compound **3**

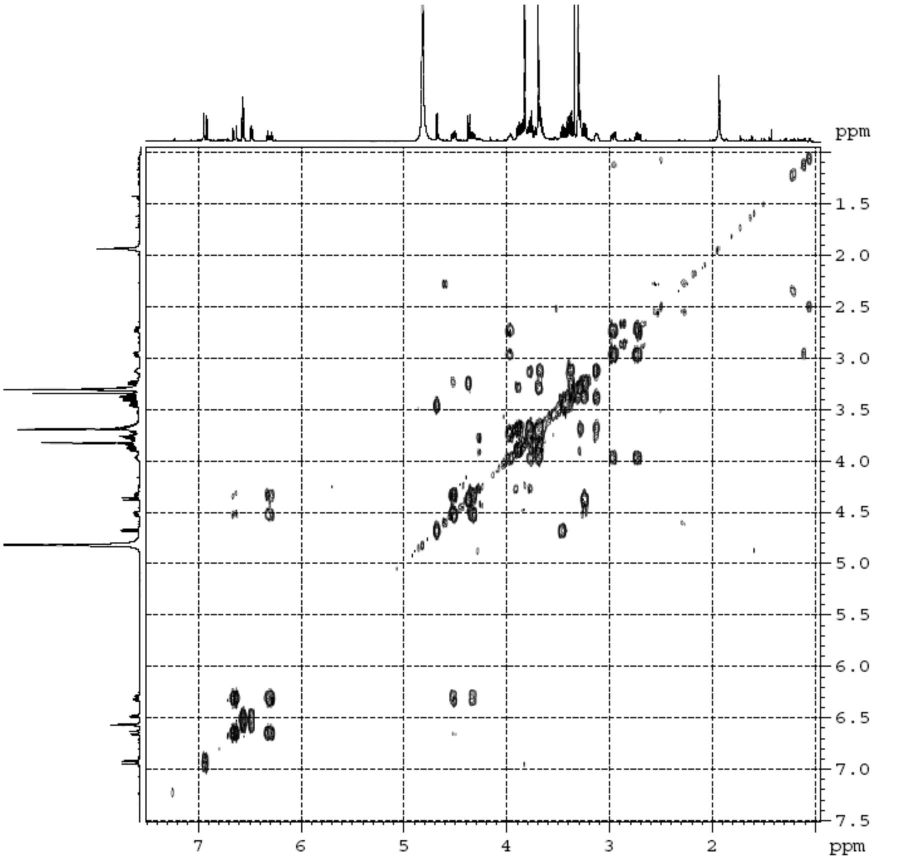

Figure S18 ^1H ^1H COSY (CD_3OD) spectrum of compound **3**

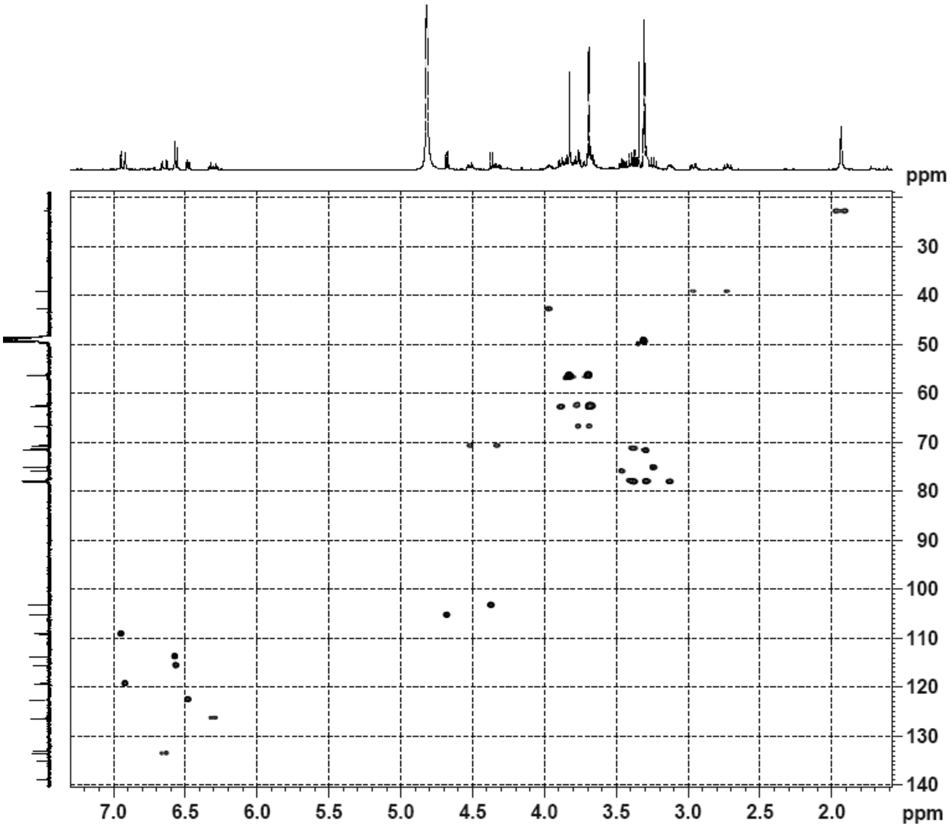

Figure S19 HSQC (CD_3OD) spectrum of compound **3**

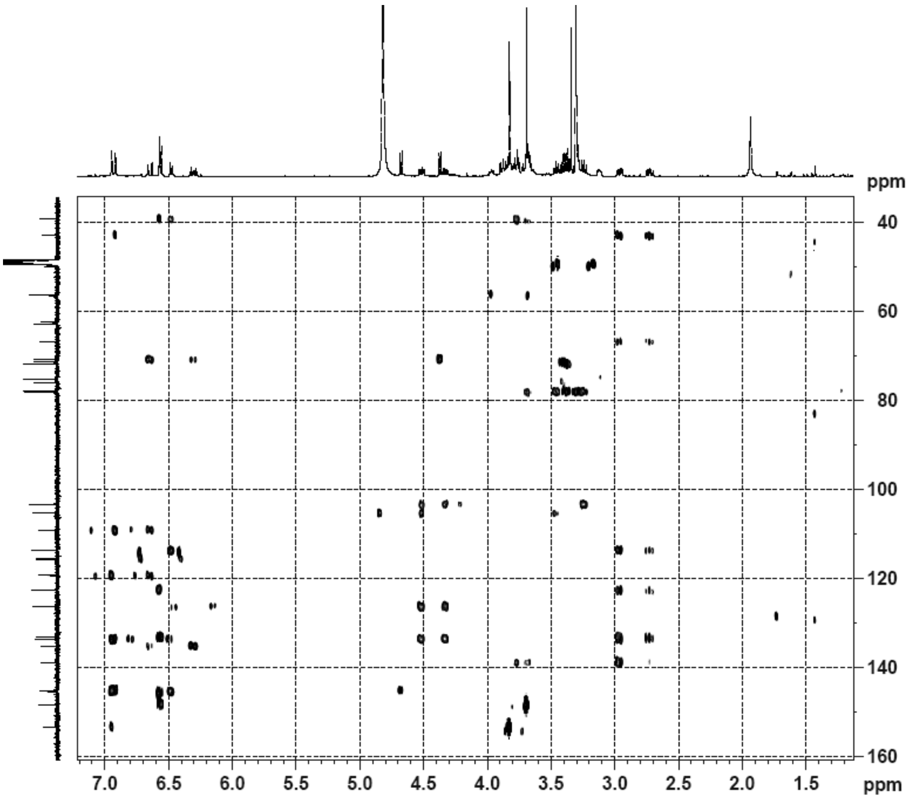

Figure S20 HMBC (CD_3OD) spectrum of compound **3**

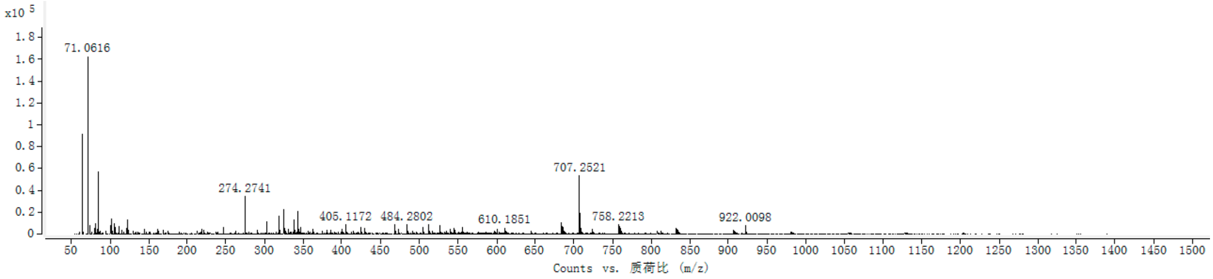

Figure S21 HRESI-TOF-MS spectrum of compound **3**

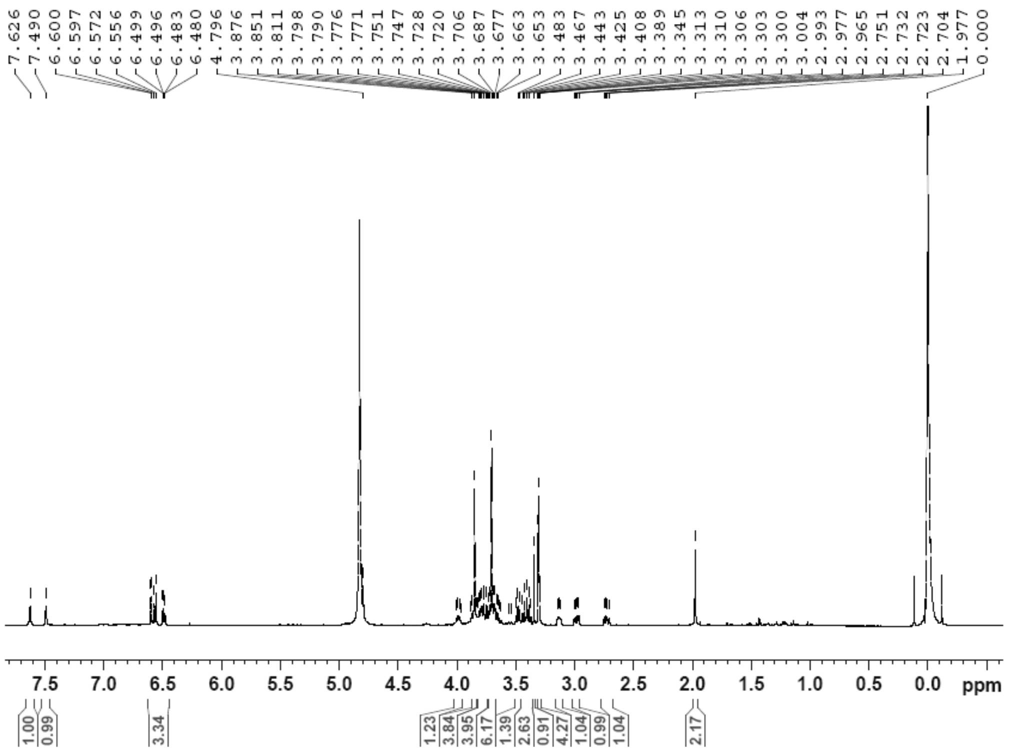

Figure S22 ^{1}H NMR (500 MHz, CD_3OD) spectrum of compound **4**

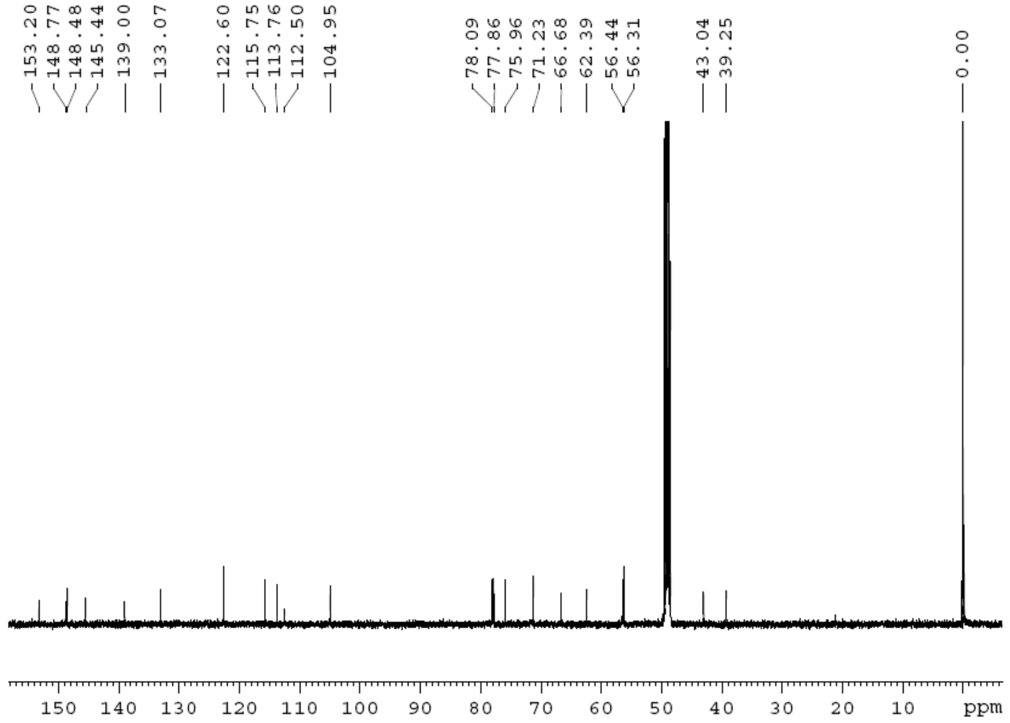

Figure S23 ^{13}C NMR (125 MHz, CD_3OD) spectrum of compound **4**

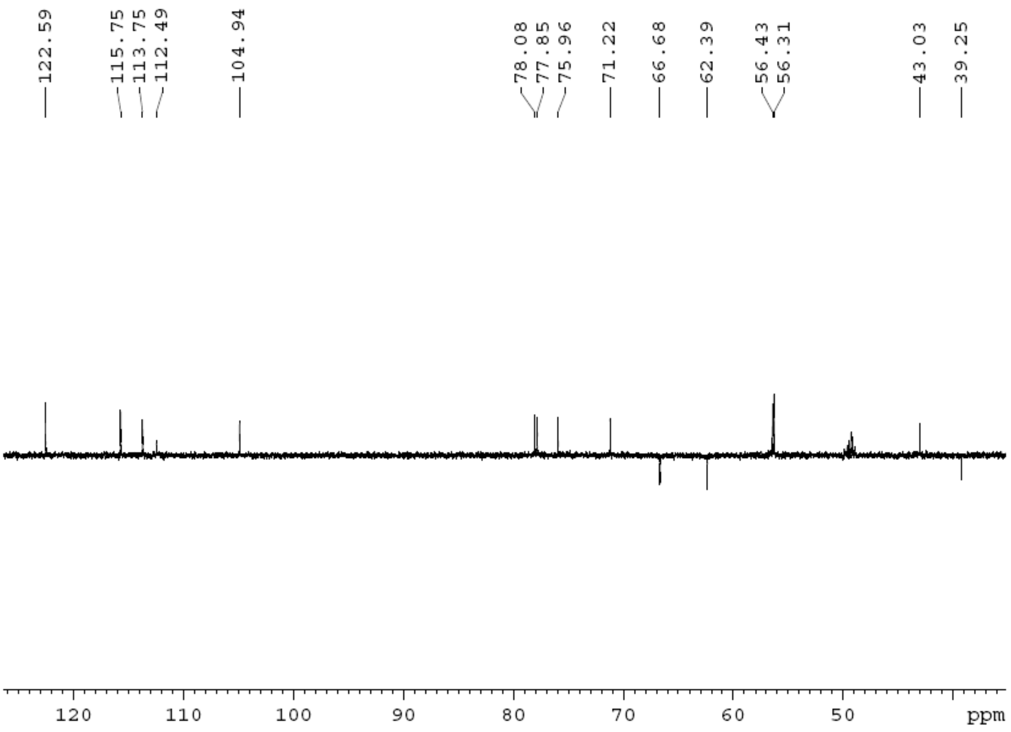

Figure S24 DEPT 135 (CD_3OD) spectrum of compound **4**

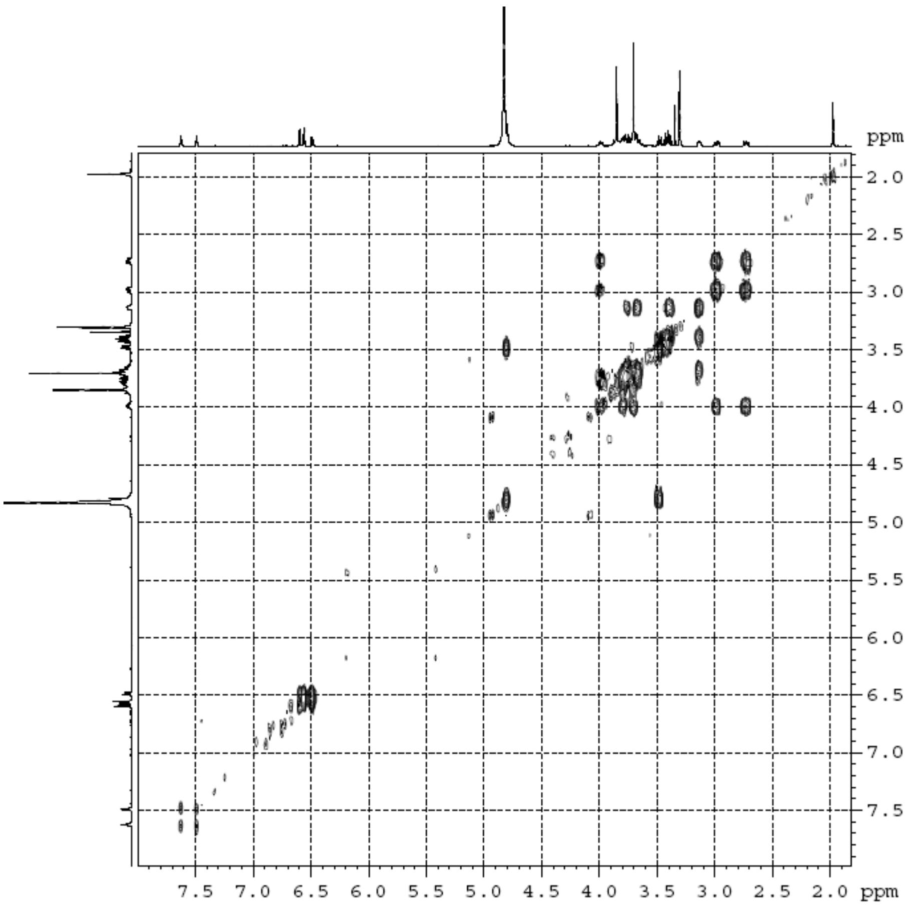

Figure S25 ^{1}H ^{1}H COSY (CD_3OD) spectrum of compound **4**

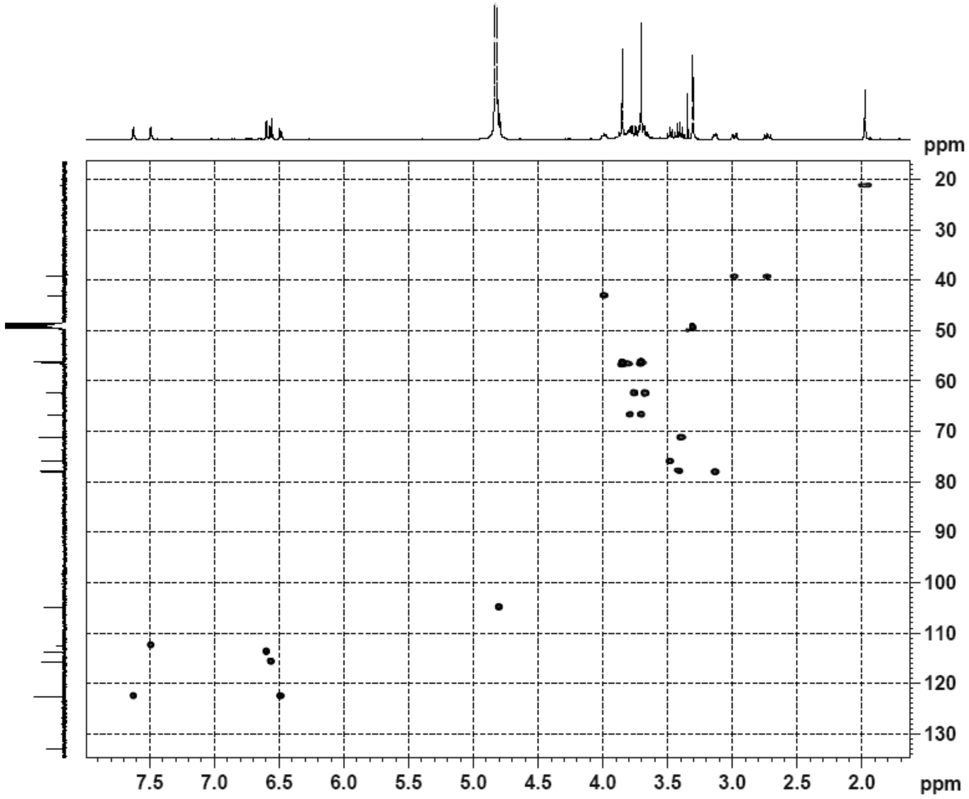

Figure S26 HSQC (CD_3OD) spectrum of compound **4**

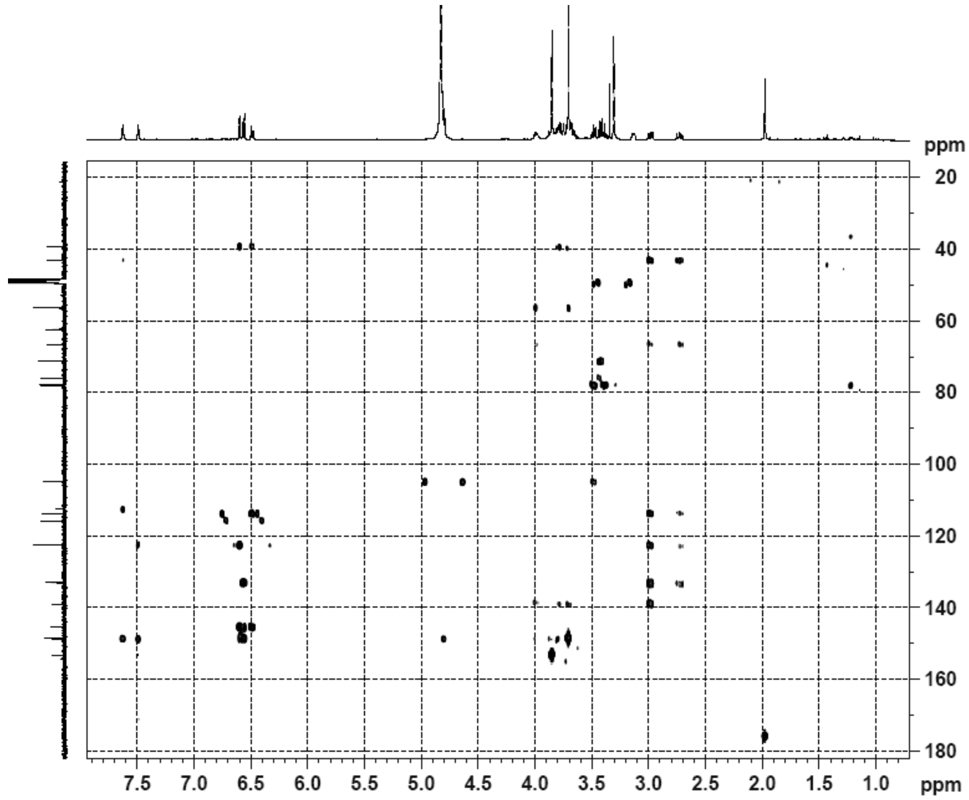

Figure S27 HMBC (CD_3OD) spectrum of compound **4**

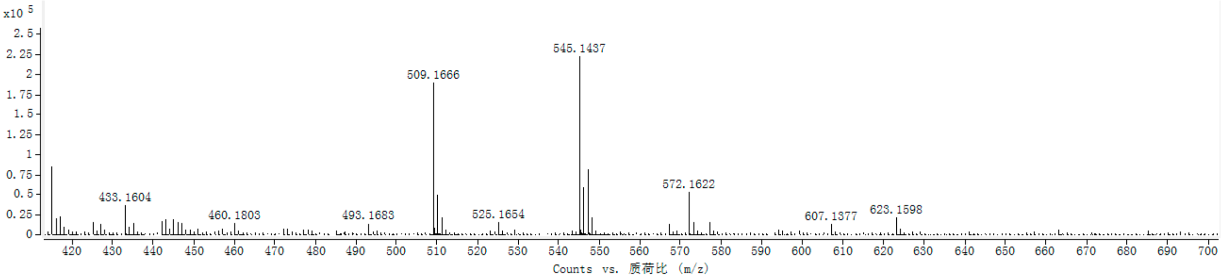

Figure S28 HRESI-TOF-MS spectrum of compound **4**

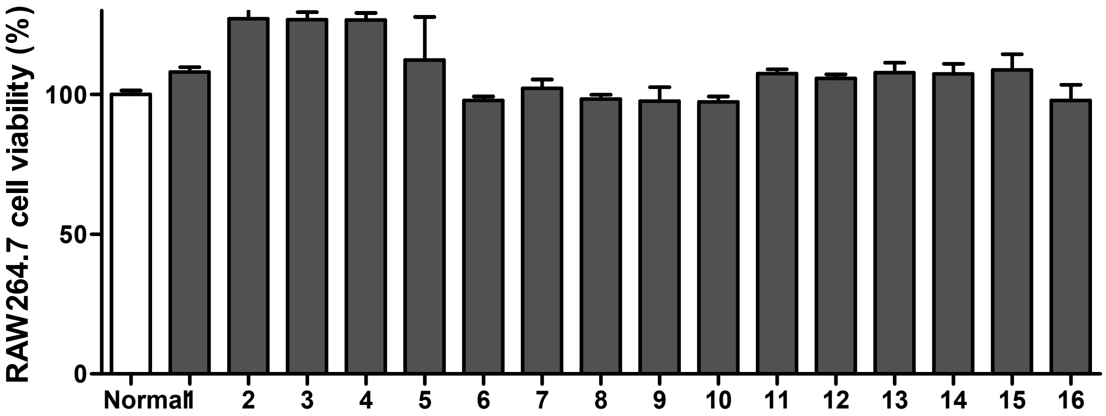

Figure S29 MTT analysis of **1–16** obtained from *Cortex Dictamni* on RAW264.7 cells.
Normal: normal group without LPS and other tested samples. Values represent the mean ± SD of four determinations. *P < 0.05; **P < 0.01; ***P < 0.001 (Differences between compound-treated group and normal group). N = 4. Final concentrations were 40μM for **1–16**, respectively.

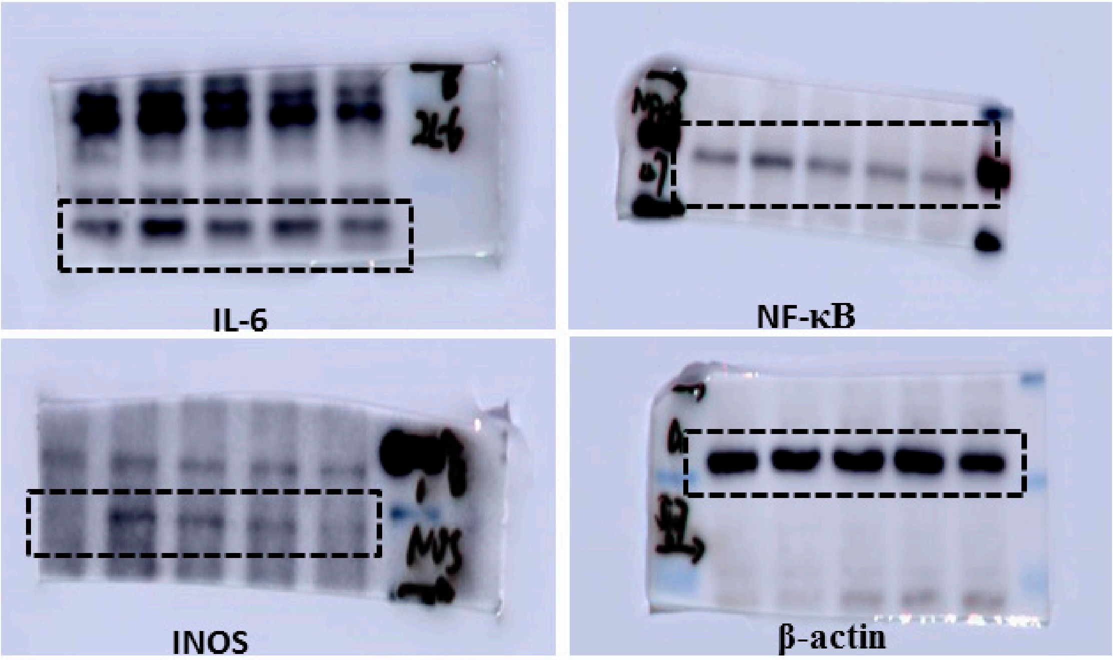

Figure S30 Raw quantification data for figure 6

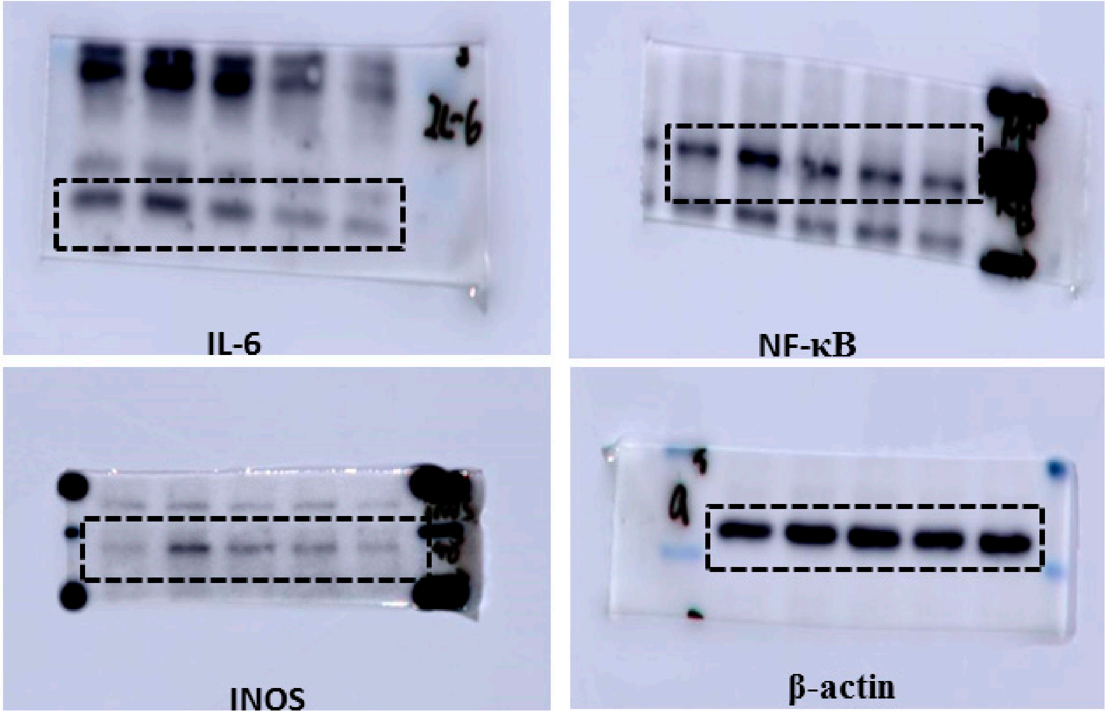

Figure S31 Raw quantification data for figure 7

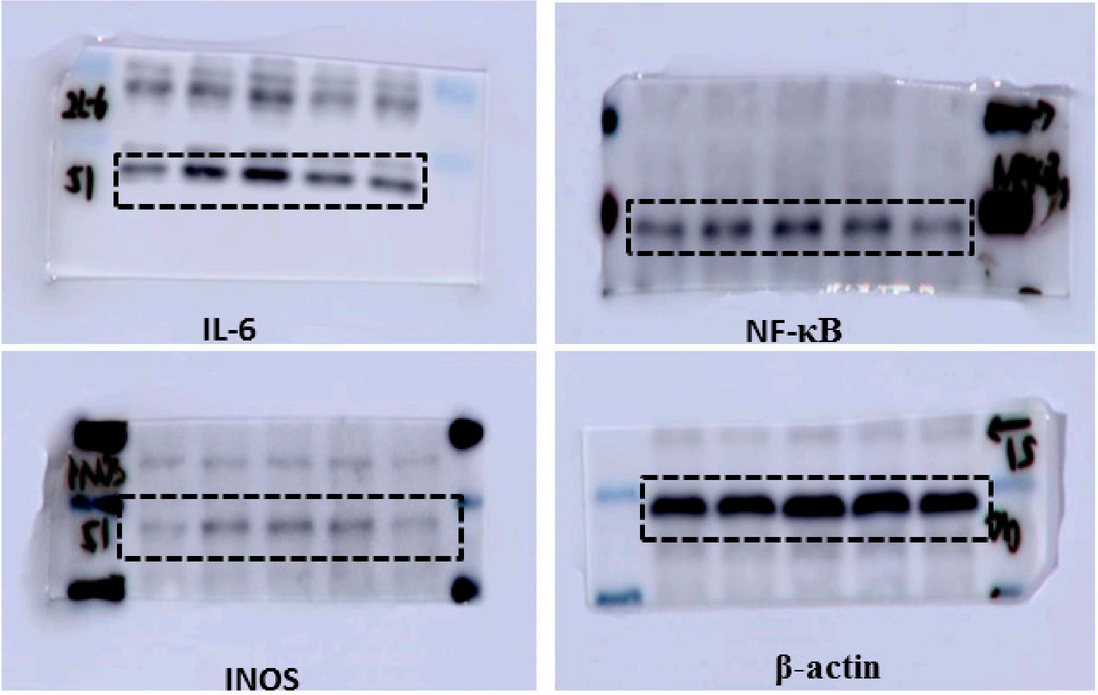

Figure S32 Raw quantification data for figure 8

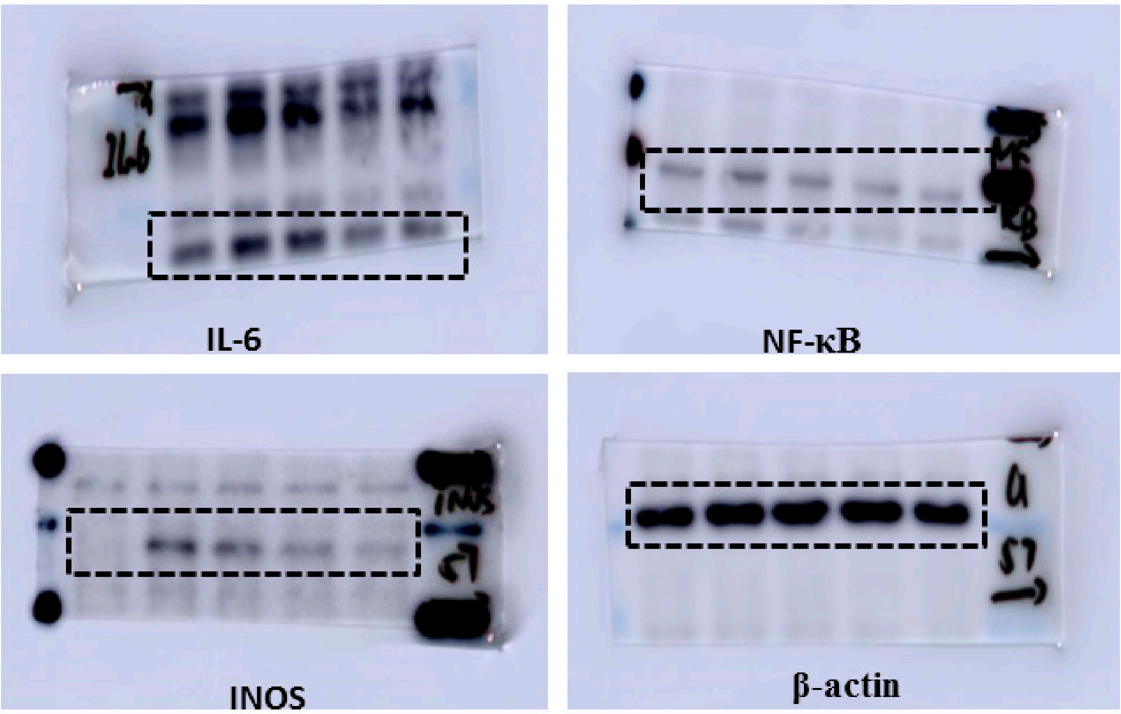

Figure S33 Raw quantification data for figure 9
